# Attitudes toward drug prescription rights: a survey of Ontario chiropractors

**DOI:** 10.1186/s12998-015-0066-7

**Published:** 2015-07-15

**Authors:** Peter Charles Emary, Kent Jason Stuber

**Affiliations:** Master of Science (MSc) Candidate, MSc Advanced Professional Practice (Clinical Sciences), Anglo-European College of Chiropractic, 13-15 Parkwood Road, Bournemouth, Dorset BH5 2DF UK; Private Practice, 201C Preston Parkway, Cambridge, ON N3H 5E8 Canada; Division of Graduate Education and Research, Canadian Memorial Chiropractic College, 6100 Leslie Street, Toronto, ON M2H 3J1 Canada

**Keywords:** Chiropractic, Attitudes, Knowledge, Drug prescription, Cross-sectional survey

## Abstract

**Background:**

Several published surveys have shown that chiropractors are generally split in their opinions regarding the right to prescribe drugs in chiropractic practice. Many of these studies have been limited by low response rates, leaving the generalizability of their findings open to question. The aim of the current study was to ascertain the general attitudes of chiropractors in Ontario, Canada toward the inclusion of drug prescription rights in their scope of practice. Relationships between these attitudes and the number of years in practice including differences in philosophical orientation were also explored.

**Methods:**

A 14-item questionnaire was developed and invitations sent via e-mail to all eligible 2,677 chiropractors in active practice registered electronically with the College of Chiropractors of Ontario in February 2015. Data were collected and analyzed using descriptive and inferential statistics.

**Results:**

960 questionnaires were completed for a 36 % response rate. The majority of respondents agreed that chiropractors should be permitted to prescribe musculoskeletal medications such as over-the-counter and prescription-based analgesics, anti-inflammatories, and muscle relaxants. Over two-thirds also felt that with limited prescriptive authority chiropractors could help reduce patients’ reliance on these types of drugs. Over three-quarters were opposed however to chiropractors having full prescribing rights. The majority indicated they recommend over-the-counter medications to acute and chronic patients to some extent in clinical practice. Nearly two-thirds perceived their knowledge of musculoskeletal medications as high or very high, while a similar proportion perceived their knowledge of drugs for non-musculoskeletal conditions to be low or very low. A majority of respondents felt that further education in pharmacology would be necessary for those in the profession wishing to prescribe medications. More recent graduates and those who espoused a broad scope of chiropractic practice were most in favour of limited prescribing rights for the profession.

**Conclusions:**

A majority of responding Ontario chiropractors expressed interest in expanding their scopes of practice to include limited drug prescription. These results together with those of other recent surveys could indicate a shift in chiropractors’ attitudes toward drug prescription rights within the profession. Further surveys and/or qualitative studies of chiropractors in other jurisdictions are still needed.

**Electronic supplementary material:**

The online version of this article (doi:10.1186/s12998-015-0066-7) contains supplementary material, which is available to authorized users.

## Introduction

In some jurisdictions in the world chiropractors can gain licensure to prescribe medications from a limited formulary of over-the-counter (OTC) and/or prescription-based medications for common musculoskeletal conditions, such as non-steroidal anti-inflammatory drugs (NSAIDs), analgesics, and muscle relaxants [1, 2]. Some within the profession feel that such prescribing rights are necessary if chiropractors are to assume the role of ‘primary spine care providers’ within the healthcare system [3, 4]. Prescribing drugs in chiropractic nevertheless remains a contentious issue and continued incorporation of these rights into the scope of chiropractic practice has major implications for the profession.

To date several published surveys [5–9] have shown that chiropractors are generally split in their opinions regarding the right to prescribe drugs in chiropractic practice. This split in opinions is most pronounced in countries where chiropractors are not currently licensed to prescribe medications. Conversely, in jurisdictions where chiropractors are licensed to prescribe from a limited formulary, such as in Switzerland, the majority perceive this right as an advantage for the profession [1, 10]. Moreover, continuing education in pharmacology is viewed by Swiss chiropractors as a necessary component of this privilege [10].

Yet despite being divided over prescribing rights in general, there is evidence to suggest that many chiropractors often recommend OTC medications to patients in practice. For example, while just over half of respondent chiropractors from surveys in Australia [5] and Oklahoma, USA [6] were supportive of prescribing rights, between 66 % and 87 % indicated they recommend non-prescription analgesics and anti-inflammatories with variable frequency to their patients. This would suggest that chiropractors that are against prescribing rights for the profession may not be entirely averse to relevant pharmaceutical use by their patients in clinical practice. As such further investigation into the frequency of OTC drug recommendation by practising chiropractors would be informative.

Contention also exists over the scope of prescription-based drug use in chiropractic practice. In New Mexico, USA, for example, chiropractors can gain licensure to prescribe from a limited formulary of musculoskeletal medications [2]. However, chiropractors in this state have also made recent attempts to expand their current formulary to include additional prescription drugs as well as drugs to be administered by injection [11] in order for chiropractors to operate as ‘primary care physicians’ [12]. Concerning the issue of full prescribing rights however, evidence from the literature suggests that chiropractors are generally opposed [5, 6, 8]. In Canada, the current knowledge and attitudes of chiropractors toward full prescribing rights is unknown and research concerning limited prescribing rights is scarce.

Questions also remain as to why the chiropractic profession is split toward prescribing rights in the first place. Some evidence suggests that this division in attitudes may be reflective of differences in philosophical orientation, with so-called ‘mixer’ chiropractors being in favour and ‘straight’ chiropractors being opposed [8]. However further research is needed in order to validate these findings, particularly within the current environment of the chiropractic profession [13, 14]. Several of the aforementioned surveys [1, 5–9] have also been limited by low response rates, leaving the generalizability of their findings open to question. As such further surveys and/or qualitative research studies are warranted in order to clarify the general attitude of chiropractors toward drug prescription in chiropractic.

The aim of this study was therefore to ascertain the general attitude (s) of chiropractors from Ontario, Canada toward the inclusion of drug prescription rights in their scope of practice. In doing so, three main areas were investigated: (i) Ontario chiropractors’ attitudes and opinions to drug prescription rights, (ii) the frequency of OTC drug recommendation by Ontario chiropractors, and (iii) Ontario chiropractors’ current knowledge of drug prescription. This study also sought to determine if there was a relationship between Ontario chiropractors’ attitudes toward drug prescription rights and (a) the number of years in chiropractic practice or employment, and (b) philosophical orientation/preferred style of practice.

## Methods

### Study design

A survey of all 2,900 chiropractors in active chiropractic practice registered through the 2014–2015 electronic directory of the College of Chiropractors of Ontario (CCO) [15] was carried out using an online, anonymous, 14-item self-administered questionnaire (see Additional file 1 for a copy of the survey instrument). Ontario chiropractors who were retired and/or who did not have an e-mail address listed with the CCO at the time of the survey were excluded. The current questionnaire was partially based on questionnaires previously used in assessing chiropractors’ opinions toward drug prescription rights [5, 8].

All qualified participants in this study were contacted via e-mail messages, at one-week intervals, up to six times over the course of six weeks. The first e-mail was a pre-notification message containing an introduction to the survey and its purpose, as well as a link to a review article on the topic of prescribing rights in chiropractic [13]. The next four e-mail messages, which included up to three reminder notifications for non-responders, were distributed through SurveyMonkey® and included a cover letter, a link to the survey instrument, as well as opt-out instructions. A final e-mail reminder was sent to non-responders on the final day before the survey was closed.

### Survey instrument

The questionnaire was divided into four sections. Section 1 consisted of four questions recorded on a 5-point Likert scale ranging from ‘strongly agree’ to ‘strongly disagree’ that focused on chiropractors’ attitudes to drug prescription rights. Section 2 consisted of two questions regarding OTC drug recommendations in chiropractic practice. Responses to both questions were recorded on a 5-point scale ranging from ‘never’ to ‘routinely.’ Section 3 contained three questions asking about the chiropractors’ current knowledge of drug prescription. Responses to the first two questions were recorded on a 5-point Likert scale ranging from ‘very high’ to ‘very low.’ Responses to the third question were recorded on a 3-point ‘yes,’ ‘no,’ or ‘don’t know’ verbal scale. Section 4 asked demographic questions including: (i) age, (ii) gender, (iii) chiropractic college of graduation, (iv) number of years in chiropractic practice or employment, and (v) chiropractic philosophical orientation/scope of practice. For this last item, respondents were asked to choose between one of three categories, as defined by McDonald et al. [8], which best described their philosophical orientation / preferred scope of practice. The three categories included: ‘broad scope’ (i.e. the often described chiropractic “mixer”), ‘middle scope,’ and ‘focused scope’ (i.e. the often described “straight” chiropractors).

### Pilot testing

An assessment of the questionnaire’s face validity [16, 17] was undertaken through peer review and a pilot study. For the pilot study a random sample of 20 chiropractors registered with the Waterloo Regional Chiropractic Society, a diverse group of currently 39 chiropractors practising within the region of Waterloo, Ontario, Canada (and representative of the target population), was used. The names of each of the 39 registered chiropractors were entered into a computer-based random number generator and the first 20 listed after randomization were selected. Each pilot study participant was asked to complete the questionnaire online, using SurveyMonkey®, and to give feedback concerning its face validity (i.e. whether or not the questionnaire adequately assessed Ontario chiropractors’ general attitudes to drug prescription rights), as well as general feedback regarding the time to complete the survey, individual item comprehension, and issues of ambiguity. There were 12 responses to the pilot study (60 % response rate) and feedback primarily consisted of comments relating to wording and clarity. All respondents affirmed the questionnaire’s face validity. This feedback was used to further revise the questionnaire, and the final survey instrument was created online and administered through SurveyMonkey®.

### Data analysis

Responses to all questions were analyzed using descriptive statistics. Central tendencies were measured as means and standard deviations for continuous data, while medians were used for ordinal data [18]. Categorical data were presented as proportions. Inferential statistics were used to investigate any differences in opinion between chiropractors who: (i) had been in practice or employed for different amounts of time (i.e. 0 to 15 years, or greater than 15 years), and (ii) had differing views regarding chiropractic philosophy/scope of practice. It was hypothesized, *a priori*, that chiropractors with a higher number of years in practice and/or who preferred a focused (or ‘straight’) chiropractic scope of practice would hold more negative views toward drug prescription rights. Relationships between these two grouping variables and the various attitudinal response variables from section 1 of the questionnaire were explored using the chi-square test of independence for nominal/categorical data [18]. In order to evaluate differences between these groups, responses to the four Likert scale items in section 1, which provided ordinal data on chiropractors’ attitudes to drug prescription rights, were collapsed and recoded as categorical data (e.g. ‘strongly agree’/ ‘agree,’ ‘neutral,’ and ‘disagree’/ ‘strongly disagree’). Statistical significance was set at *p* < 0.05, and all data analysis was carried out using SPSS (Statistical Program for the Social Sciences, © IBM SPSS Statistics, Version 20).

### Ethical considerations

Prior to data collection, ethics approval (E67/05/15) was obtained through the Anglo-European College of Chiropractic Research Ethics Sub-Committee. The Research Ethics Board Secretariat for Health Canada was also contacted and further ethics review in Canada was deemed not necessary due to the nature of the research being undertaken in this study. All data collected for this study was recorded anonymously and stored securely in a password protected electronic database.

## Results

After removing duplicate and invalid e-mail addresses from the 2014–2015 CCO directory, the questionnaire was sent to 2,847 chiropractors in Ontario, representing more than two-thirds (68.0 %) of all chiropractors in active practice registered with the CCO at the time of the survey (February 2, 2015 to February 27, 2015). One hundred and seventy questionnaires were automatically returned as undeliverable due to change of recipient e-mail addresses (*n* = 77) or those previously having opted out of receiving SurveyMonkey® surveys (*n* = 93). Of the remaining 2,677 questionnaires that were sent, completed questionnaires were received from 960 respondents (35.9 % response rate), representing the views of nearly one-quarter of the profession in Ontario at the time.

Table 1 provides demographic comparisons between the study sample and the general population of Ontario chiropractors. With respect to philosophical orientation, nearly one-third (31.7 %) of respondents classified themselves as practising within a ‘broad scope’ of chiropractic practice, over half (54.8 %) were ‘middle scope,’ and the remaining 13.4 % (128/952) of respondents identified themselves as ‘focused scope’ chiropractors.

**Table 1:** Demographic comparison of study respondents versus all Ontario chiropractors in active practice at the time of the survey

Variable	Study respondents	All Ontario chiropractors^a^
	(*n* = 960)	(*n* = 4,189)
Mean (SD) age, years	44 (11)	44 (11)
Gender		
• Male, %	70 (670/951)	64 (2679/4187)
• Female, %	30 (281/951)	36 (1508/4187)
College of graduation		
• CMCC, %	72 (689/952)	73 (3033/4178)
• USA, %	26 (245/952)	26 (1100/4178)
• Outside USA, %	2 (15/952)	1 (35/4178)
• UQTR, %	0 (3/952)	0 (9/4178)
Mean (SD) years in practice	17 (11)	15 (12)

*SD* = standard deviation, *CMCC* = Canadian Memorial Chiropractic College, *USA* = United States of America, *UQTR* = Université de Québec à Trois Riviéres

^a^Values derived from demographic data provided by the College of Chiropractors of Ontario (as of December 5, 2014)

**Fig. 1:**
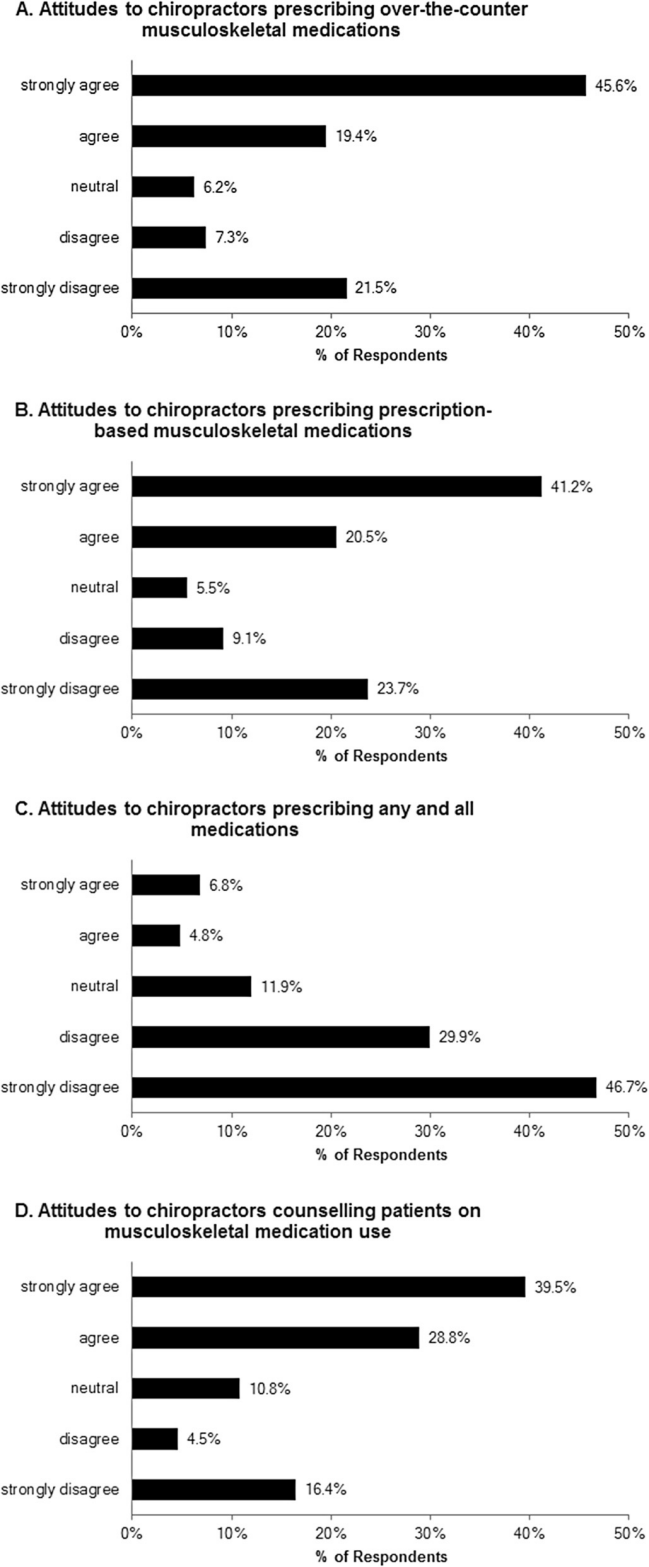
Ontario chiropractors’ attitudes to drug prescription rights. (**a**) Responses regarding attitudes to chiropractors prescribing over-the-counter musculoskeletal medications (*n* = 958), median value = ‘agree.’ (**b**) Responses regarding attitudes to chiropractors prescribing prescription-based musculoskeletal medications (*n* = 952), median value = ‘agree.’ (**c**) Responses regarding attitudes to chiropractors prescribing any and all medications (*n* = 958), median value = ‘disagree.’ (**d**) Responses regarding attitudes to chiropractors counselling patients on musculoskeletal medication use (*n* = 955), median value = ‘agree’

Ontario chiropractors’ attitudes to drug prescription rights obtained from section 1 of the questionnaire are summarized in Fig. 1. The majority of respondents were in favour of incorporating limited drug prescription rights within their scope of practice. Nearly two-thirds (65.0 %) were in agreement that chiropractors should be able to gain an expanded scope to allow for prescription of OTC medications for common musculoskeletal conditions (Fig. 1a). Similarly, the majority (61.7 %) agreed that chiropractors should be able to gain an expanded scope of practice to allow for the prescription of a limited number of prescription-based musculoskeletal medications (Fig. 1b). Respondents were not in favour of chiropractors having full prescribing rights, with a large majority (76.6 %) disagreeing that chiropractors should be able to gain an expanded scope to allow for the prescription of any and all medications, including controlled substances (Fig. 1c). Finally, a majority (68.3 %) of respondents agreed that if given limited prescriptive authority chiropractors could play a role in counselling patients against overuse and over-reliance on medications for musculoskeletal conditions (Fig. 1d).

**Fig. 2:**
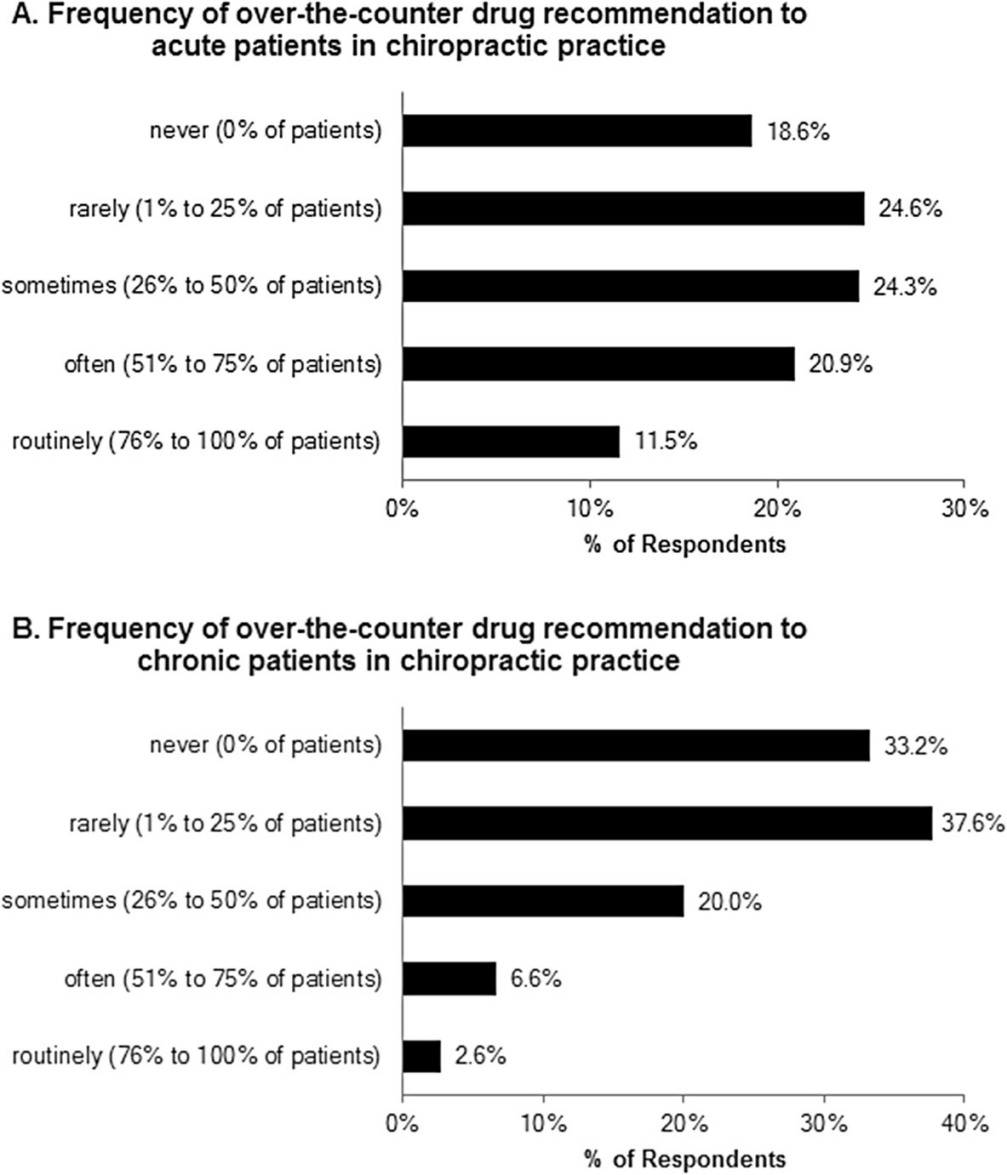
Frequency of over-the-counter drug recommendation by Ontario chiropractors. (**a**) Responses regarding the frequency of over-the-counter drug recommendation to acute patients in clinical practice (*n* = 955), median value = ‘sometimes.’ (**b**) Responses regarding the frequency of over-the-counter drug recommendation to chronic patients in clinical practice (*n* = 957), median value = ‘rarely’

Responses to section 2 of the questionnaire asking about the frequency of OTC drug recommendation are shown in Fig. 2. Overall, the majority of respondents indicated that they recommend OTC drugs to patients to some extent in clinical practice. Respondents also suggested OTC medications more frequently to acute patients (Fig. 2a) than chronic patients (Fig. 2b).

**Fig. 3:**
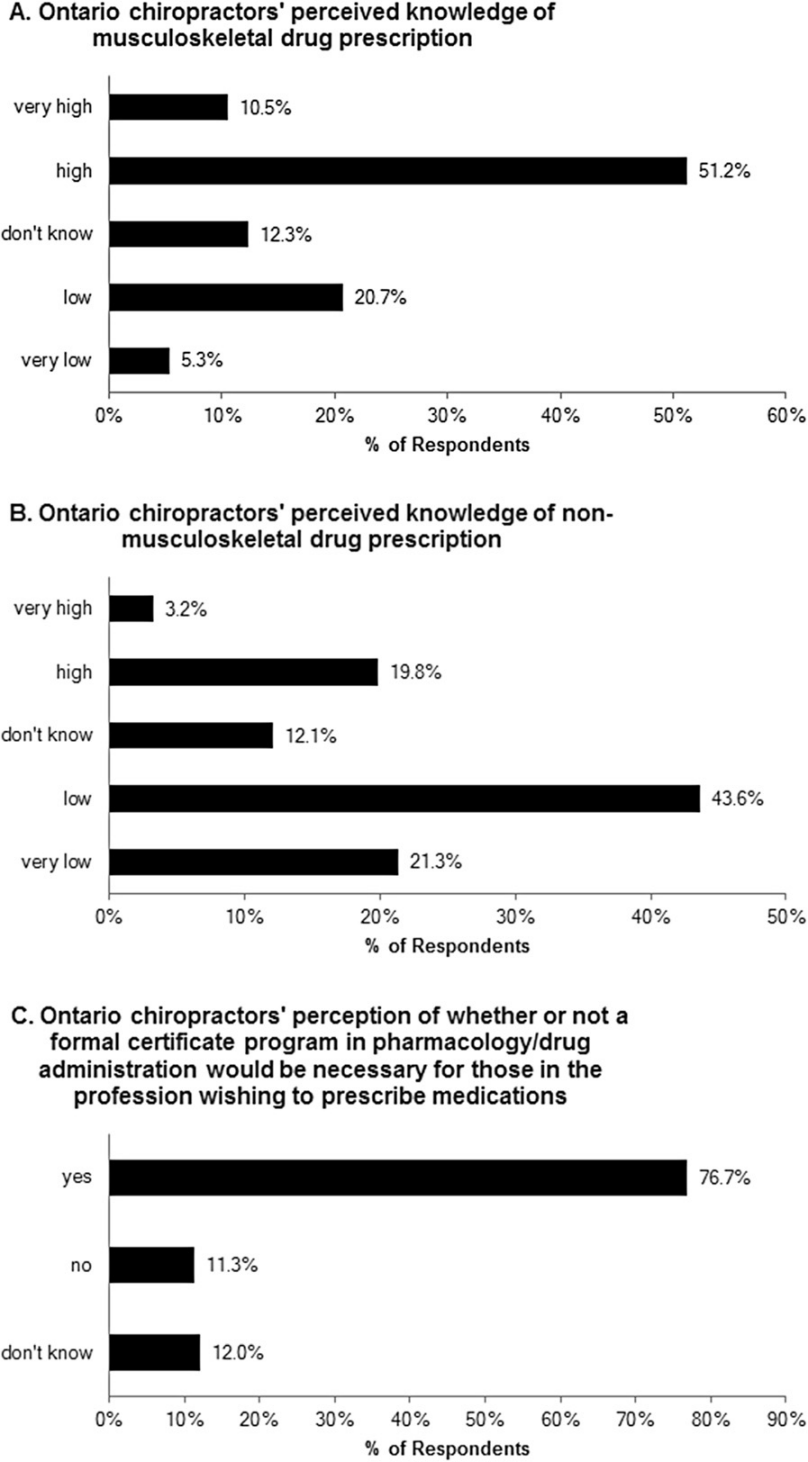
Ontario chiropractors’ current perceived knowledge of drug prescription. (**a**) Responses regarding Ontario chiropractors’ perceived knowledge of musculoskeletal drug prescription (*n* = 956), median value = ‘high.’ (**b**) Responses regarding Ontario chiropractors’ perceived knowledge of non-musculoskeletal drug prescription (*n* = 955), median value = ‘low.’ (**c**) Responses regarding Ontario chiropractors’ perception of whether or not a formal certificate program in pharmacology/drug administration would be necessary for those in the profession wishing to prescribe medications (*n* = 952), modal value = ‘yes’

Responses to section 3 of the questionnaire exploring current knowledge of drug prescription are summarized in Fig. 3. Respondents were generally confident regarding their perceived knowledge towards prescribing musculoskeletal medications (Fig. 3a), but less so for drugs used in treating non-musculoskeletal conditions (Fig. 3b). A large majority (76.7 %) of respondents also felt that completion of a formal postgraduate certificate program in pharmacology/drug administration should be required for those in the profession wishing to prescribe medications (Fig. 3c).

Comparisons between Ontario chiropractors’ attitudes to drug prescription rights and the number of years in practice are displayed in Table 2. A statistically significant greater proportion of respondents with less than 15 years’ experience agreed that Ontario chiropractors should be able to prescribe OTC and prescription-based musculoskeletal medications compared to those with more than 15 years’ experience. Respondents with more than 15 years’ experience also disagreed significantly more so than those with less than 15 years’ experience regarding the idea that chiropractors with limited prescriptive authority could counsel patients on musculoskeletal medication use. With respect to the issue of full prescribing rights, no statistically significant difference in opinion was found between chiropractors who had been in practice or employed for different amounts of time.

**Table 2:** Comparison of Ontario chiropractors’ attitudes to drug prescription rights based on the number of years in practice

Years in practice	Agree %	Neutral %	Disagree %
Attitudes to chiropractors prescribing OTC MSK medications^a^			
0 to 15 years	69.8 (369/529)	5.5 (29/529)	24.8 (131/529)
>15 years	59.3 (248/418)	6.9 (29/418)	33.7 (141/418)
Attitudes to chiropractors prescribing prescription-based MSK medications^b^			
0 to 15 years	65.8 (347/527)	4.9 (26/527)	29.2 (154/527)
>15 years	56.5 (234/414)	6.0 (25/414)	37.4 (155/414)
Attitudes to chiropractors prescribing any and all medications^c^			
0 to 15 years	12.5 (66/529)	11.7 (62/529)	75.8 (401/529)
>15 years	10.8 (45/418)	12.2 (51/418)	77.0 (322/418)
Attitudes to chiropractors counselling patients on MSK medication use^d^			
0 to 15 years	71.8 (379/528)	12.3 (65/528)	15.9 (84/528)
>15 years	64.2 (267/416)	8.9 (37/416)	26.9 (112/416)

OTC = over-the-counter, MSK = musculoskeletal

^a^χ^2^_2df_ = 11.24; *P* = 0.004

^b^χ^2^_2df_ = 8.55; *P* = 0.014

^c^χ^2^_2df_ = 0.68; *P* = 0.714

^d^χ^2^_2df_ = 18.07; *P* < 0.001

Comparisons between Ontario chiropractors’ attitudes to drug prescription rights and differences in philosophical orientation are displayed in Table 3. Among ‘broad scope’ respondents an overwhelming majority were in agreement that Ontario chiropractors should be able to gain an expanded scope to allow for prescription of OTC and prescription-based analgesics, NSAIDs, and muscle relaxants. Relatively few of the ‘focused’ group respondents held the same opinion. ‘Middle scope’ chiropractors were also in favour of limited prescribing rights, although to a lesser extent than their ‘broad scope’ colleagues supporting the idea of being able to prescribe OTC and prescription-based musculoskeletal medications. Similarly, a large majority of ‘broad scope’ and ‘middle scope’ respondents agreed that if given limited prescriptive authority, chiropractors could play a role in counselling patients against overuse and over-reliance on drugs commonly prescribed for musculoskeletal conditions. In contrast, less than one-quarter of ‘focused scope’ respondents supported this idea. Regarding full prescribing rights, nearly one-quarter of the ‘broad scope’ group agreed that Ontario chiropractors should be able to gain an expanded scope allowing for the prescription of any and all medications, including controlled substances, however the proportion of ‘middle scope’ and ‘focused scope’ chiropractors who similarly agreed was considerably lower.

**Table 3:** Comparison of Ontario chiropractors’ attitudes to drug prescription rights based on philosophical orientation

Philosophical orientation	Agree %	Neutral %	Disagree %
Attitudes to chiropractors prescribing OTC MSK medications^a^			
Broad scope	93.0 (281/302)	1.3 (4/302)	5.6 (17/302)
Middle scope	62.2 (324/521)	8.8 (46/521)	29.0 (151/521)
Focused scope	11.0 (14/127)	6.3 (8/127)	82.7 (105/127)
Attitudes to chiropractors prescribing prescription-based MSK medications^b^			
Broad scope	90.9 (271/298)	1.7 (5/298)	7.4 (22/298)
Middle scope	57.0 (296/519)	7.3 (38/519)	35.6 (185/519)
Focused scope	12.6 (16/127)	6.3 (8/127)	81.1 (103/127)
Attitudes to chiropractors prescribing any and all medications^c^			
Broad scope	23.9 (72/301)	17.3 (52/301)	58.8 (177/301)
Middle scope	6.1 (32/522)	10.7 (56/522)	83.1 (434/522)
Focused scope	5.5 (7/127)	3.9 (5/127)	90.6 (115/127)
Attitudes to chiropractors counselling patients on MSK medication use^d^			
Broad scope	90.0 (271/301)	4.3 (13/301)	5.6 (17/301)
Middle scope	66.2 (344/520)	14.2 (74/520)	19.6 (102/520)
Focused scope	23.8 (30/126)	11.9 (15/126)	64.3 (81/126)

OTC = over-the-counter, MSK = musculoskeletal

^a^χ^2^_4df_ = 296.23; *P* < 0.001

^b^χ^2^_4df_ = 254.18; *P* < 0.001

^c^χ^2^_4df_ = 89.81; *P* < 0.001

^d^χ^2^_4df_ = 221.24; *P* < 0.001

## Discussion

The main finding of this study was that the majority of Ontario chiropractors who responded to this survey were in favour of incorporating limited drug prescription rights into their scope of practice. Nearly two-thirds agreed that chiropractors should be permitted to prescribe OTC and prescription-based analgesics, NSAIDs, and muscle relaxants. Almost 70 % also felt that with limited prescriptive authority chiropractors could help counsel patients against overuse and over-reliance on these types of medications. This level of support for chiropractic prescribing rights is in contrast to that of previously published research which has shown that the profession has generally been divided on this topic [13]. A majority (55.2 %) of respondents from a recent survey of North American chiropractic students [19] were also not in favour of expanding the chiropractic scope of practice to include drug prescription. The current study’s findings are nevertheless in line with those of several recent unpublished surveys where between 55 % and nearly 80 % of respondents supported the idea of chiropractors prescribing musculoskeletal medications [20, 21] (B. Haig, Chief Executive Officer, Ontario Chiropractic Association; personal communication, 3 November 2014). These conflicting results in the literature reiterate the need for further investigation in order to clarify the general attitude of chiropractors internationally towards drug prescription in the profession.

In spite of this, evidence from two of the aforementioned surveys including that in the present study indicate that there may be a growing interest among Ontario chiropractors towards limited chiropractic prescribing rights. For instance, in surveys involving members of the Ontario Chiropractic Association from 2007 and 2011 (B. Haig, Chief Executive Officer, Ontario Chiropractic Association; personal communication, 3 November 2014), increasing majorities (55 % and 61 %) of respondents respectively were in favour of chiropractors prescribing anti-inflammatory and/or analgesic medications. An even greater majority favouring limited prescribing rights in the present study suggests that there may be a possible shift in chiropractors’ attitudes toward drug prescription rights occurring within the profession in Ontario.

In Switzerland, where chiropractors already have limited prescribing rights, the profession is more united regarding drug prescription in chiropractic [1, 10] and is strongly integrated and accepted by the medical community [22]. As such Swiss chiropractors have cultural authority within the musculoskeletal domain. For instance, chiropractic is among one of five government-recognized medical professions in Switzerland (i.e. human medicine, chiropractic medicine, veterinary medicine, dentistry, and pharmacology), and chiropractic treatment is fully covered under the Swiss national health insurance program [22]. If chiropractors in other countries wish to gain drug prescription privileges however, there are numerous implications to consider. These would include, but are not limited to, the need for additional education and training for chiropractors in pharmacology and toxicology, necessary regulatory and legislative changes, consideration of legal and ethical issues, and increases to chiropractic malpractice/liability insurance coverage [13, 23].

Concerning the issue of pharmacology education, the current study found that Ontario chiropractors were quite confident regarding their perceived knowledge towards prescribing musculoskeletal medications. In fact, nearly two-thirds of respondents indicated that their current knowledge of these drugs was ‘high’ or ‘very high.’ Interestingly nearly equal numbers perceived their current knowledge of drugs for non-musculoskeletal conditions as ‘low’ or ‘very low.’ The first finding is surprising given that the basic chiropractic educational curriculum contains only 12 h of coursework in pharmacology [24]. A possible explanation is that over 72 % of respondents in the current study graduated from the Canadian Memorial Chiropractic College where students presently receive 30 h of training in pharmacology and toxicology [25]. Although this number of hours in pharmacology education is above the World Health Organization standards for chiropractic, students in other healthcare professions such as dentistry complete an average of almost 70 h [26] and chiropractic students in Switzerland take over 80 h in pharmacology at the University of Zürich (C.K. Peterson, personal communication, 18 January 2015). Regardless of how confident Ontario chiropractors might be regarding their perceived knowledge towards musculoskeletal medications, further undergraduate and/or post-graduate education and training would be necessary in order to competently prescribe these types of medications in clinical practice. In fact, this view was supported by a large majority of respondents in the current study as over three-quarters felt that completion of a formal postgraduate certificate program in pharmacology/drug administration should be required for those in the profession wishing to prescribe medications. Currently, chiropractors in New Mexico, USA must complete a two-year postgraduate Master of Science degree in ‘Advanced Clinical Practice’ [4, 27] before they can obtain a license to prescribe from the limited chiropractic formulary in that state [2]. This postgraduate program offers further training in pharmacology [27] and could serve as a model for the profession, particularly in other jurisdictions where chiropractic prescribing rights are being considered.

Despite evidence to suggest that chiropractors in Ontario and elsewhere are interested in gaining limited prescriptive privileges, a large majority of respondents in the current study did not favour the idea of chiropractors having full prescribing rights. More than three-quarters disagreed that chiropractors should be able to gain an expanded scope to allow for the prescription of any and all medications, including controlled substances. This finding is consistent with those of previous surveys of chiropractors from Australia [5], the United States [6], and North America [8] where respondents were generally opposed to chiropractors writing drug prescriptions for non-musculoskeletal conditions. This is also in accordance with the views of those in the medical profession whose members would likely oppose such an expansion to the chiropractic scope of practice as well [23]. On the other hand, if chiropractors would focus their scope to treating spine-related/musculoskeletal conditions there is evidence to suggest that medical doctors would support limited prescription privileges for the chiropractic profession [22, 23, 28]. Some chiropractors in New Mexico, USA have nevertheless attempted to expand their existing formulary to beyond a limited number of medications, and this has been met by opposition from both the medical and chiropractic professions in that state [11].

Another finding of the current study was that a large number of Ontario chiropractors in this survey tend to recommend OTC drugs to their patients. For instance, when asked how often they suggested non-prescription analgesic and NSAID medications to acute and chronic patients in clinical practice, 81 % and 67 % of respondents indicated that they did so to some extent respectively. These non-prescription drug utilization rates are comparable to those of other published studies of practising chiropractors [1, 5, 6, 10, 22], and are congruent with current evidence-based guidelines [29–31]. This nevertheless suggests that several chiropractors in Ontario are making treatment recommendations that are outside of their current legislative scope of practice [32]. Arguably however, this study’s findings indicate the need to align the chiropractic scope of practice with current scientific evidence as well as individual practitioner behaviour. The findings of this study also suggest that many chiropractors support OTC drug use in clinical practice no matter what their personal stance is on prescribing rights for the profession. For at least some of these chiropractors this points to a disconnect between traditional chiropractic philosophy (i.e. non-drug, non-surgical health care) and once again, actual practice behaviour. Interestingly, the remaining respondents in the present study indicated that they would ‘never’ recommend OTC analgesics and NSAIDs to their patients. It is unclear if these participant responses were based on individual chiropractic philosophical orientation, or simply that these clinicians felt that OTC drug recommendation was outside the scope of chiropractic practice.

When exploring possible reasons for why some chiropractors have differing views toward drug prescription, an association was found in this study between respondents’ opinions and the number of years in practice. For instance, chiropractors who had practised for 15 years or less were significantly more in favour of musculoskeletal drug prescription rights versus those with greater than 15 years’ experience. This difference in opinion between the two groups could possibly reflect slightly differing views toward evidence-based practice. For example, several clinical guidelines endorse the use of mild analgesics and/or anti-inflammatories in the management of various musculoskeletal conditions [29–31]. Yet some literature suggests that more experienced practitioners are less likely to view research evidence as valuable or necessary in their day-to-day clinical practice [33–35]. The current study did not directly inquire about respondents’ attitudes to evidence-based practice, so it is unclear whether this characteristic actually influenced respondent opinions toward drug prescription in the survey. The differences may have once again been based more on respondents’ philosophical orientation and/or attitudes toward current chiropractic scope of practice. Regardless, the majority of respondents from both groups (greater than 15 years versus 15 years or less in practice) still favoured the idea of limited prescribing rights for chiropractors despite their overall practice experience.

As for philosophical orientation, this study showed that there was a strong relationship between this variable and Ontario chiropractors’ attitudes to drug prescription rights. For instance, almost all of the ‘broad scope’ respondents in the survey were in favour of Ontario chiropractors gaining prescriptive rights for treating musculoskeletal conditions, whereas very few of the ‘focused scope’ group felt the same way. These findings are consistent with those from the study by McDonald et al. [8] where more than three-quarters of broad scope respondents supported limited prescribing rights compared to less than one-fifth of focused scope chiropractors who similarly agreed. The majority of ‘middle scope’ respondents in the current study also favoured musculoskeletal prescribing rights. The majority (53.5 %) of middle scope respondents in the McDonald et al. [8] survey supported limited chiropractic prescribing rights as well, but to a lesser extent than those in the current study. Where broad and middle scope chiropractors from the present study disagreed was regarding full prescribing rights; nearly one-quarter of respondents in the broad scope group agreed that chiropractors should be permitted to write prescriptions for any and all medications while virtually none in the middle scope group held a similar view. Akin to the situation in New Mexico, USA, however, this attitude of favouring full prescribing rights for chiropractors by some broad scope respondents is in contrast to the general view held by many others in the profession [5, 6, 8].

There may be a middle ground concerning chiropractic prescribing rights where some level of agreement within the profession could be reached. For instance, evidence from the literature including results from the current study suggest that among chiropractors who hold favourable views toward drug prescription, prescription privileges limited to within a musculoskeletal scope of practice would be preferred [5, 6, 8]. A large majority of respondents in the current study also agreed that with limited prescriptive authority chiropractors could advise patients against overusing analgesic and anti-inflammatory medications. Evidence to support this notion can be found in Switzerland where chiropractors tend to prescribe medications significantly less so than asked for by their patients [10]. With the over-prescription of drugs such as opioids in countries like the United States [36], the ability of chiropractors to counsel patients on musculoskeletal drug use is something that all members of the profession should be interested in, regardless of philosophical orientation. In fact, a large majority of broad and middle scope chiropractors in the current study supported this potential role for the profession. Focused scope respondents did not, however, as less than one-quarter similarly agreed. As such, these findings along with those of others [8] suggest that complete consensus on the topic of chiropractic prescribing rights will likely remain elusive for the profession given the philosophical views traditionally held by this minority (13 % in the current study) group of chiropractors. However in light of the fact that physiotherapists are interested in and are gaining limited drug prescription rights in some countries [37, 38], it is imperative that the remaining majority of the chiropractic profession continues this discussion. Further surveys and/or qualitative studies of chiropractors’ opinions toward gaining prescription privileges in these and other jurisdictions would be timely. In Canada, the results of the current study may be taken to other provinces in order to complete a nationwide survey. If the same findings are confirmed elsewhere, it would argue for a national campaign to reform the chiropractic scope of practice acts across the country.

### Limitations

This study has some limitations. First, the overall response to the survey was relatively low (36 %) thus raising the likelihood of non-response/exclusion bias in the results [18]. However, the number of responses (*n* = 960) obtained in this study was higher than those of other published surveys on chiropractic prescribing rights [1, 5–10], and when comparing demographic characteristics the sample appears to be representative of the general population of practising chiropractors in Ontario (see Table 1). Nevertheless, a 64 % non-response rate suggests that these survey results should be interpreted with caution as respondents’ views toward drug prescription rights obtained may not be generalizable to those of all Ontario chiropractors.

A second limitation of this study is that it excluded retired chiropractors and those not on the electronic 2014–2015 directory of the CCO. As these groups represented the minority (32 %) of all licensed chiropractors in Ontario at the time of the survey, this was felt to be less of an issue. Nonetheless, there is a risk that retired chiropractors and/or those who did not have an e-mail address listed with the CCO may have held systematically different views toward drug prescription rights compared to chiropractors listed on the electronic CCO directory.

Thirdly, chiropractors’ attitudes to drug prescription rights were measured in this study using a closed-answer format only. Although good for aggregating data from large study populations, the disadvantage to using this survey method is that it does not allow participants to expand upon responses or offer alternative viewpoints [17], and this would have prevented any ‘richness’ to the responses in the current study. On the other hand, open-answer questions take longer to complete which can dissuade participants from responding [17]. These questions can also be laborious (and expensive) to analyze qualitatively [17], particularly with large data sets, and was beyond the scope of the current study.

## Conclusions

This study revealed that a majority of Ontario chiropractors were in favour of incorporating limited drug prescription rights into their scope of practice, were generally confident regarding their knowledge of musculoskeletal medications, and tended to recommend OTC drugs such as mild analgesics and/or anti-inflammatories to patients to some extent in clinical practice. However, respondents did not favour the idea of chiropractors having full prescribing rights, were not confident in their knowledge of drugs for non-musculoskeletal conditions, and felt that further education and training in pharmacology should be necessary for those in the profession wishing to prescribe medications. Those who had been in practice for less than 15 years favoured musculoskeletal prescribing rights more so than chiropractors with more than 15 years’ experience; however the overall majority in both groups still favoured limited prescribing rights for the profession. As for philosophical orientation, the majority of broad and middle scope respondents in this study also favoured limited chiropractic prescribing rights, whereas those in the focused scope group did not. Further surveys and/or qualitative studies of chiropractors in other jurisdictions are needed in order to validate these findings.

## Additional file

Additional file 1:
**Survey instrument.**

## Abbreviations

CCO: College of Chiropractors of Ontario
CMCC: Canadian Memorial Chiropractic College
MSK: Musculoskeletal
NSAIDs: Non-steroidal anti-inflammatory drugs
OTC: Over-the-counter
SD: Standard deviation
SPSS: Statistical Program for the Social Sciences
UQTR: Université de Québec à Trois Riviéres
USA: United States of America
