# Supplementary material for: Attitudes toward drug prescription rights: a survey of Ontario chiropractors
Source: Chiropr Man Therap. 2015 Jul 15;23:22. doi: 10.1186/s12998-015-0066-7 (PMC4502931; doi:10.1186/s12998-015-0066-7)
Supplement: Additional file 1: — Survey instrument. [file 12998_2015_66_MOESM1_ESM.docx]

**Section 1. Ontario chiropractors’ attitudes toward drug prescription rights**

In some jurisdictions in the world chiropractors are licensed to prescribe from a limited formulary of over-the-counter and/or prescription-based medications for common musculoskeletal conditions (e.g. analgesics, non-steroidal anti-inflammatories, and muscle relaxants). Some within the profession feel that such prescribing rights are necessary if chiropractors are to assume the role of ‘primary spine care providers’ within the healthcare system. In at least one of these jurisdictions chiropractors are also interested in expanding this privilege to include additional prescription drugs as well as drugs to be administered by injection, in order for chiropractors to operate as ‘primary care physicians.’

Please indicate your level of agreement with the following statements:

1. Chiropractors should be able to gain an expanded scope of practice to allow for prescription of over-the-counter medications for common musculoskeletal conditions (e.g. simple analgesics, non-steroidal anti-inflammatories, and/or muscle relaxants).

___strongly agree

___agree

___neutral

___disagree

___strongly disagree

1. Chiropractors should be able to gain an expanded scope of practice to allow for the prescription of a limited number of prescription-based analgesics, non-steroidal anti-inflammatories, and/or muscle relaxants.

___strongly agree

___agree

___neutral

___disagree

___strongly disagree

1. Chiropractors should be able to gain an expanded scope of practice to allow for the prescription of any and all medications, including controlled substances (e.g. antibiotics, anti-hypertensives, anti-depressants, corticosteroids, etc.).

___strongly agree

___agree

___neutral

___disagree

___strongly disagree

1. The legal right to prescribe certain drugs to patients in chiropractic practice would include the right of *un*-prescribing these same drugs.

Therefore if given limited prescriptive authority, for example, chiropractors could play a role in counselling patients against overuse and over-reliance on medications such as analgesics, non-steroidal anti-inflammatories, and muscle relaxants.

___strongly agree

___agree

___neutral

___disagree

___strongly disagree

**Section 2. Frequency of over-the-counter drug recommendation in chiropractic practice**

Current evidence-based guidelines endorse the use of mild analgesics and non-steroidal anti-inflammatories in the management of musculoskeletal conditions such as low back pain, neck pain, and headache.

1. Regardless of whether or not you agree with drug prescription rights in chiropractic, how often do you suggest over-the-counter medications, such as Tylenol or Advil, to acute patients in clinical practice?

___never (0% of patients)

___rarely (1% to 25% of patients)

___sometimes (26% to 50% of patients)

___often (51% to 75% of patients)

___routinely (76% to 100% of patients)

1. Regardless of whether or not you agree with drug prescription rights in chiropractic, how often do you suggest over-the-counter medications, such as Tylenol or Advil, to chronic patients in clinical practice?

___never (0% of patients)

___rarely (1% to 25% of patients)

___sometimes (26% to 50% of patients)

___often (51% to 75% of patients)

___routinely (76% to 100% of patients)

**Section 3. Ontario chiropractors’ current knowledge of drug prescription**

The basic chiropractic educational curriculum contains approximately 12 hours of coursework in pharmacology, although the actual number of course hours may vary from educational institution to institution. At the Canadian Memorial Chiropractic College, for example, students currently receive 30 hours of undergraduate education in pharmacology and toxicology.

1. How do you perceive your current knowledge regarding the prescription of medications for common musculoskeletal conditions (i.e. analgesics, non-steroidal anti-inflammatories, and muscle relaxants)?

___very high

___high

___don’t know

___low

___very low

1. How do you perceive your current knowledge regarding the prescription of medications for non-musculoskeletal conditions (i.e. antibiotics, anti-hypertensives, anti-depressants, etc.)?

___very high

___high

___don’t know

___low

___very low

1. Do you feel that completion of a postgraduate certificate program in pharmacology / drug administration should be required for chiropractors wishing to prescribe medications?

___yes

___no

___don’t know

**Section 4. Demographics and scope of practice**

1. What is your age?

______

1. What is your gender?

___Male

___Female

1. What chiropractic college did you graduate from?

___Canadian Memorial Chiropractic College

___Université de Québec à Trois Riviéres

___Other – USA

___Other – outside USA

1. How many years have you been a chiropractor?

______

1. Which of the following best describes your chiropractic philosophical orientation / preferred scope of practice?

___Broad scope

___Middle scope

___Focused scope

Definitions:

*Broad scope* – allows for a wide array of manual and other clinical procedures for diagnosing and treating both symptoms and neuromusculoskeletal conditions. Some in this camp would prefer to include minor surgery, obstetrics, and prescribing medications.

*Middle scope* – tends to combine spinal and/or extremity adjusting with other conservative treatment and diagnostic procedures.

*Focused scope* – emphasizes the detection and adjustment of vertebral subluxations to restore normal nerve activity to musculoskeletal and visceral tissues. Some in this camp oppose therapeutic modalities, extremity adjusting, and diagnostic procedures.
